# Investigating Health Risk Environments in Housing Programs for Young Adults: Protocol for a Geographically Explicit Ecological Momentary Assessment Study

**DOI:** 10.2196/12112

**Published:** 2019-01-10

**Authors:** Benjamin F Henwood, Brian Redline, Eldin Dzubur, Danielle R Madden, Harmony Rhoades, Genevieve F Dunton, Eric Rice, Sara Semborski, Qu Tang, Stephen S Intille

**Affiliations:** 1 Suzanne Dworak-Peck School of Social Work University of Southern California Los Angeles, CA United States; 2 Department of Preventive Medicine Keck School of Medicine University of Southern California Los Angeles, CA United States; 3 College of Engineering Northeastern University Boston, MA United States; 4 College of Computer and Information Science and Bouvé College of Health Sciences Northeastern University Boston, MA United States

**Keywords:** homelessness, ecological momentary assessment, experience sampling, social environment, qualitative research, geography

## Abstract

**Background:**

Young adults who experience homelessness are exposed to environments that contribute to risk behavior. However, few studies have examined how access to housing may affect the health risk behaviors of young adults experiencing homelessness.

**Objective:**

This paper describes the Log My Life study that uses an innovative, mixed-methods approach based on geographically explicit ecological momentary assessment (EMA) through cell phone technology to understand the risk environment of young adults who have either enrolled in housing programs or are currently homeless.

**Methods:**

For the quantitative arm, study participants age 18-27 respond to momentary surveys via a smartphone app that collects geospatial information repeatedly during a 1-week period. Both EMAs (up to 8 per day) and daily diaries are prompted to explore within-day and daily variations in emotional affect, context, and health risk behavior, while also capturing infrequent risk behaviors such as sex in exchange for goods or services. For the qualitative arm, a purposive subsample of participants who indicated engaging in risky behaviors are asked to complete an in-depth qualitative interview using an interactive, personalized geospatial map rendering of EMA responses.

**Results:**

Recruitment began in June of 2017. To date, 170 participants enrolled in the study. Compliance with EMA and daily diary surveys was generally high. In-depth qualitative follow-ups have been conducted with 15 participants. We expect to recruit 50 additional participants and complete analyses by September of 2019.

**Conclusions:**

Mixing the quantitative and qualitative arms in this study will provide a more complete understanding of differences in risk environments between homeless and housed young adults. Furthermore, this approach can improve recall bias and enhance ecological validity.

**International Registered Report Identifier (IRRID):**

DERR1-10.2196/12112

## Introduction

### Background

Risk environment has been defined as the space—whether social or physical—in which factors external to a person interact to increase the chances of certain health risk behaviors [1-3]. Young adults between the ages of 18 and 25 years old, sometimes referred to as transition-aged youth, who experience homelessness live in an unstable and sometimes chaotic risk environment that has resulted in high rates of substance use and sexually transmitted infections, including HIV [4-7]. For example, Noell et al found the incidence of sexually transmitted infections in a homeless adolescent population to be as high as 17% and the prevalence and incidence of certain infections to be 10 to 12 times higher than those found in the same age group among the general population [7]. Homelessness services programs that provide housing to young adults have the capacity to change their risk environment, and housing might serve as a protective factor by providing safe, independent living arrangements that can alleviate stress related to being homeless [8,9]. Housing programs may also expose young adults to positive social influences, because street-based peers have been associated with risk behaviors in this population [10,11]. However, these programs may also change the contextual factors that influence health risk behaviors in unforeseen or unexpected ways; for example, being placed in a housing program may increase social pressures from network members who are still experiencing homelessness and need a place to stay or the privacy afforded by housing could permit misuse and sale of drugs or easier engagement in risky sex [12,13]. To date, there has been limited investigation of young adults with a history of homelessness who have enrolled in housing programs [14] or differences in the risk environment between those experiencing homelessness and those who have moved into housing programs. Furthermore, past studies typically relied on self-report methods that are burdened by recall bias [1,3,5].

### Objectives

**Figure 1:**
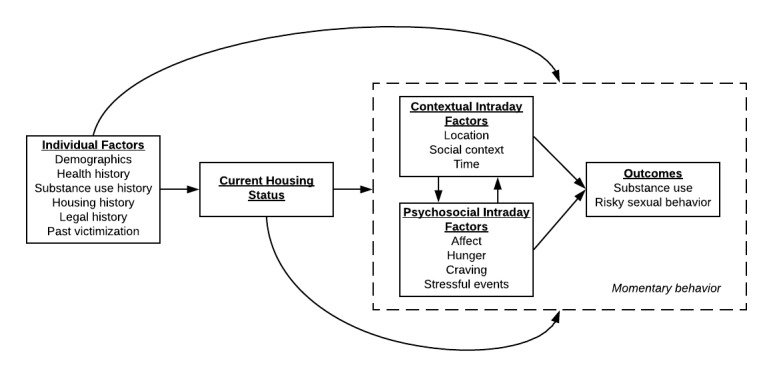
Log My Life conceptual framework.

The Log My Life (LML) study seeks to fill this gap in the literature by developing an innovative, mixed-methods approach using geographically explicit ecological momentary assessments (GEMA) to understand the risk environment of young adults who have either enrolled in housing programs or are currently experiencing homelessness. GEMA is considered the gold standard for capturing valid intensive longitudinal self-report information that is embedded in important contextual factors and can be used to understand and predict health risk behaviors [15-19]. The conceptual model for this study (see Figure 1) is based on prior research, showing that within-day variation in various psychosocial characteristics (eg, mood and substance use craving) affects both drug use and sexually risky behavior [20-25]. We hypothesize that access to housing for individuals experiencing homelessness will affect where, when, and with whom they spend time daily [26-28]. Furthermore, we hypothesize that these contextual factors can influence HIV risk and within-day psychosocial characteristics [6,21,29-34], which in turn could impact one’s ability to access or maintain stable housing.

This paper describes the protocols of the LML study and highlights innovative aspects of its design, including the use of geospatial data, ecological momentary assessments (EMA), dynamic social contexts, and in-depth interviews to assess the built and social context and psychosocial factors influencing risky health behavior. We also present preliminary recruitment progress to date and describe additional avenues of potential inquiry.

## Methods

### Design Overview

Leveraging the widespread use of smartphone technology, including among homeless populations [35,36], this study uses a mixed-methods, prospective longitudinal design to recruit young adults who are in either of the following sampling frames: (1) enrolled in a housing program (eg, transitional housing and permanent supportive housing) or (2) currently homeless. For the quantitative arm, participants in both sampling frames complete a baseline questionnaire and are observed for 1 week by responding to repeated momentary surveys prompted and administered through a smartphone app developed for this study. A 7-day period was chosen to have adequate power (ie, 8 prompts per day for a total of 56 within-subject observations) to detect within-subject changes over time and to capture variation in behavior based on the day of the week. Both EMAs and daily diaries are prompted, and responses are used to explore within-day and daily variations in emotional affect, context, and risk behavior, while also capturing infrequent risk behaviors such as sex in exchange for goods or services. The geospatial location of the participant is also continuously recorded for the monitoring period, to the degree permitted by the phone. For the qualitative arm, a purposive subsample of participants who indicated engaging in risky behaviors are asked to complete an in-depth qualitative interview using an interactive, personalized geospatial map rendering of EMA responses as an elicitation device. Similar methods have been used to better understand contextual factors that influence tobacco use [15]. Mixing the quantitative and qualitative arms in this study provides a more complete understanding of differences in risk environments between homeless and formerly homeless young adults. Study protocols were approved by the institutional review board at the University of Southern California.

### Participants

Participants include young adults residing in Los Angeles County who either have or are experiencing homelessness. Approximately 200 participants are currently being recruited from 11 agencies that run permanent supportive or transitional living housing programs as well as from shelter sites and drop-in facilities serving youth experiencing homelessness. To investigate the various contextual mechanisms that could explain risk behaviors among young adults, a power analysis was conducted to determine that we need approximately 100 young adults in housing programs and 100 young adults who remain homeless. In both sampling frames, individuals are eligible to participate if they can be interviewed in English, can read and understand smartphone items in English without assistance, and are willing to provide written informed consent. To be included in the housed sample, young adults must be aged between 18 and 25 years and residing in a housing program that serves homeless young adults. Individuals as old as 27 years are allowed to participate if they entered the housing program before the age of 25 years. Young adults are considered to be part of the unhoused sample if they are aged between 18 and 25 years and meet the McKinney-Vento Homeless Assistance Act [37] definition of homelessness that specifies lack of a fixed, regular, and adequate nighttime residence.

### Recruitment

Young adults are being recruited through flyers and informational sessions held at housing programs or drop-in facilities. Study staff members have or will recruit youth at 6 permanent housing programs, 9 transitional living programs, and 6 drop-in facilities across Los Angeles County. Young adults recruited at permanent or transitional living programs are considered to be eligible if they are enrolled in the housing program and fit inclusion criteria. During informational sessions at drop-in sites, youth complete a self-administered screener on an electronic tablet that indicates whether they meet the eligibility criteria for homelessness or are enrolled in a housing program.

### Procedures

Upon enrollment, participants receive an iPad (Apple, USA) to complete a self-administered questionnaire via a secure Web-based platform. Due to the potentially sensitive nature of the questions, the questionnaire is administered using computer-assisted self-interviewing techniques. These baseline meetings last approximately 45 to 75 min and include a questionnaire with 2 components: one that assesses demographics and historical experiences and another that explores participants’ social network (subsequently described). Participants then have the option to use a study-provided phone, usually a third-generation MotoG (Motorola, USA) smartphone that has an unlimited data plan, or their personal smartphone if they own an Android-based phone that is compatible with the study smartphone app. Youth who agree to use their personal phone receive an additional US $10 to offset the cost of cellular data. Throughout the study period, momentary and daily surveys are prompted using a custom software app for smartphones running the Android operating system (Google, USA). Participants can earn up to US $130 for completing the main study components, but some incentives are task-based (ie, each participant’s total incentive amount is driven by compliance with prompted EMAs and daily diaries). Research staff members assist the participant in setting up the smartphone app during baseline meetings, and each participant completes 1 practice EMA and daily diary demonstration. During setup, participants specify normal sleep and wake times, so they do not receive prompts outside their typical waking hours.

For the next 7 days, participants complete EMAs on the smartphone app. EMAs allow repeated collection of real-time data, eliminate the need for retrospective recall, and are particularly well suited for examining episodic behavior that may be affected by context such as substance use [19,38-40]. Participants receive 7 to 8 prompts per day, depending on the number of waking hours. Participants are prompted randomly during 30-min windows separated by 2-hour intervals. For example, if a participant is normally awake from 9 am to 11 pm, 1 EMA survey will be triggered randomly between 9:30 am and 10 am, 11:30 am and 12 pm, 1:30 pm and 2 pm, and so on. The app delivers a push notification with a chime or vibration, if the phone is not set to silent. Participants are instructed to stop their current activity and complete a short EMA survey on their phone. This process requires about 1 to 3 min. If no entry is made, the app emits up to 3 push notifications at 3-min intervals (3, 6, and 9 min after the first prompt). Each EMA survey becomes inaccessible 10 min after the initial prompt, unless the participant is answering the survey. Participants are instructed to ignore signals that occur during an incompatible activity (eg, driving, sleeping, or bathing). EMA surveys contain skip logic to minimize burden on the participant while optimizing the quality of data received in each brief survey. A minimum of 15 items appear at each EMA prompt. A maximum of 31 items appear in the event that a participant reports all permutations of risk behavior and social context. Soliciting multiple EMA entries per day has been shown to be acceptable in previous studies with youth and adults [41-44] and had been pilot tested for this study.

Starting on the second day of the observation period and continuing for 7 consecutive days, participants are asked to complete a daily diary in which they reflect on their behavior during the previous day. Participants can self-initiate and complete the daily diary at any point during the day, but they are also automatically prompted to do so if the daily diary has not already been completed. Participants select 3 times throughout the day (eg, 9 am, 12 pm, and 2 pm) to receive reminder prompts to complete the daily diary. Reminders do not deploy within 15 min of EMA prompting windows to avoid conflict between surveys. Although momentary data capture can be considered an improvement over self-report methods, there is still a need to include daily assessments as a complement to EMA as we continue to build these methods [45]. Capturing assessments in both momentary and daily methods will provide an opportunity to evaluate how well daily diaries replicate momentary data. Use of daily methods permits more flexibility in how questions are asked. In addition, daily diaries are useful for querying infrequent risk behaviors that would be unnecessary to ask multiple times per day (eg, frequency of sex), as is often done with studies deploying EMA methods. To date, studies comparing collection methods have found close approximate aggregated ratings of behavior or affect between daily and momentary collection [46], but daily collection provides a much better representation of real-time experiences than longer recall methods [47,48]. Responses on both EMA and daily diaries are encrypted, wirelessly uploaded after each entry, and stored on a server accessible to the research team for compliance monitoring.

During the monitoring week, study personnel contact participants by phone twice to check on progress, encourage compliance, and address any technical issues. Participants can also email, call, or text a study helpline number any time they have issues or questions. Google Voice is used to maintain a record of calls, texts, and emails as well as allow multiple staff members to address concerns and mask their own personal phone numbers. After the monitoring period is completed, participants meet with the study staff to complete a 30-min exit appointment, during which they respond to additional questionnaires, receive compensation (calculated based on their compliance), and return the study phone and charger if borrowed.

Study personnel invite a purposive subsample of study participants (n=30) who (1) indicate high-risk behavior (eg, hard drug use and frequent alcohol or marijuana use) during their observation week or during their lifetime and (2) display adequate compliance (ie, 70% or greater) on EMA and daily diaries to participate in an additional in-depth, 45- to 60-min qualitative interview. This interview is used to explore dynamic socioenvironmental factors that affect health risk behaviors and how youth navigate risky environments. The structured open-ended interview also uses an interactive, personalized geospatial map rendering of EMA responses that are generated through the smartphone’s built-in location-finding system as an elicitation device, similar to a method proposed by McQuoid et al [15]. These interviews are conducted in private rooms at drop-in sites or housing programs. Audio of the session are recorded and transcribed using a professional service, and participants receive US $30.

### Measures

#### Baseline and Exit Questionnaires

##### Baseline Questionnaire

The baseline questionnaire addresses factors and characteristics shown to be related to housing stability among youth [9,49-51], including demographics (eg, age, sex, gender, race and ethnicity, education, income, and current and past employment), physical and mental health conditions, lifetime and recent drug and alcohol use, trauma history, homelessness history, life skill development, and emotional regulation. Participant mental health is assessed with the Patient Health Questionnaire [52,53], General Anxiety Disorder Scale [54], Primary Care PTSD Screener [55], Difficulties in Emotion Regulation Scale Short Form [56], Perceived Stress Scale [57], and the COPE scale [58], all of which have been validated with young adult populations. Mental health diagnoses, suicidality, and care engagement are also assessed. Participants rate their current physical health, list their chronic illnesses, assess sleep impairment, and complete a checklist about difficulties in health care access. Items to address youth sexual history and sex-related HIV risk behaviors are based on the Centers for Disease Control and Prevention Youth Risk Behavior Survey’s sexual behaviors subsection [59-61], a validated tool that details past 90-day sexual behaviors of adolescents. Items in this portion of the baseline questionnaire also assess HIV and sexually transmitted infection testing, status, and treatment and are based on previous studies by this research team [26,62]. Youth provide a detailed account of lifetime and past 30-day substance use, including type of drug, typical quantity and frequency of consumption, and route of administration (eg, injected, swallowed, or smoked). Probable alcohol or other substance use disorder is determined with the CAGE substance abuse screening tool [63,64], a short, validated assessment that examines substance use issues among individuals aged 16 years or above. In addition to risky behaviors, positive youth development, such as ability to communicate, look forward, manage money, or practice self-care, is measured with the Ansell-Casey Life Skills assessment [65,66].

The baseline questionnaire additionally focuses on participants’ historical life experiences such as duration of homelessness, foster care involvement, and justice system involvement, also based on measures used in other studies with homeless youth [26,62]. Childhood trauma is assessed with the University of California, Los Angeles Post-Traumatic Stress Disorder Reaction Index for the Diagnostic and Statistical Manual of Mental Disorders-IV [67], and participants also reflect on past experiences with trauma in their community, including incidents of robbery, violent assault, intimate partner violence, or other experiences perceived as discrimination. Participants are asked to recall their past experiences with police and gangs and discuss their access to guns. In addition to past experiences, youth complete the Stress on the Streets Checklist [68] to describe their current level of stress with their living environment and the Food Insecurity Scale [69] to assess their typical access to food. Finally, youth comment on their knowledge of housing options, engagement with meaningful activities, and access to supportive services (eg, drop-in center, shelter, and mental health counseling).

##### Social Networks

To assess social networks, participants also complete a short egocentric social network inventory (based on REALYST [70]; also refer to the study by Burt [71]). Participants initially identify 5 people (commonly referred to as *alters* in social network analysis [72]) that they interact with most frequently (eg, friend from the street, family member, romantic partner, or caseworker). Then, participants respond to questions about each named individual’s characteristics, including the alter’s gender, age, sexual orientation, race and ethnicity, nature of relationship, frequency of contact, substance use belief and behaviors, sexual beliefs and behaviors, and whether the alter is a source of advice or support.

##### Exit Questionnaire

During the exit meeting, participants complete an additional computer-assisted questionnaire that includes items about life skill development [65] and if housed, their current housing experience based on items from the Housing Experience Survey [73]. During the exit meeting, youth have an opportunity to describe any events that may have made the observation week atypical and reflect on their experience in the study, such as whether EMA prompts or time spent responding to surveys interfered with their daily life, caused any stress or anxiety, or altered their typical behavior. Participants also indicate the extent to which they felt comfortable answering items honestly and accurately and whether they generally had a negative or positive experience using the smartphone app.

#### Ecological Momentary Assessment and Daily Diaries

##### Ecological Momentary Assessments

Surveys prompted by the phone during each EMA query momentary positive and negative affect, hunger, significant events (eg, involved in a physical fight), and further details and contextual factors concerning alcohol use, other drug use, and temptation to drink or use drugs. These items have been successfully applied in other EMA studies of affect and substance use [21,22,25,74-78]. EMA also prompts request information about the participant’s social context (subsequently described), and each response is time stamped. A complete list of EMA items and their response options along with sample screenshots can be found in Multimedia Appendices 1 and 2.

##### Daily Diaries

Daily diaries capture risk behaviors of the previous day and infrequent behavior that may be missed by EMAs. In daily diaries, participants respond to items that aim to provide more in-depth details about any drug use events (eg, quantity and mode of use) or sexual encounters that occurred during the previous day (eg, partner’s gender, nature of relationship with partner, and use of a condom). Participants also reflect on their sleep behavior during the past evening, including the duration, location, and quality. Items in the daily diary, available in Multimedia Appendix 3, were adapted from the Youth Risk Behavior Surveillance System survey [61] and tools developed by our team in previous studies [62].

##### Dynamic Social Context

The names of the 5 individuals with whom the participant interacts most (ie, alters) elicited from the baseline questionnaire are entered into the smartphone app during setup. The app stores each entry, subsequently adding the variables as responses into the social context items of each EMA and daily diary. At the start of each EMA (see Multimedia Appendix 1), participants select which alters (if any) they interacted with during the past 2 hours; if any alters were present, participants indicate whether alcohol, tobacco, or other drugs were consumed. A participant may also specify the presence of other individuals, as desired. If a participant reports interacting with someone other than 1 of the 5 alters, an additional follow-up question gathers data on the relationship between that person and the participant. Although EMA items assess the presence of alters and other individuals at a given moment, daily diaries assess with which specific alters the participant may have consumed alcohol or drugs, if at all (see Multimedia Appendix 3). Relevant alter-level characteristics from the baseline questionnaire (eg, alter uses illicit substances) can be used to generate risk profiles corresponding to the social context of each assessed interaction. For example, prompt-level data may contain a sum of the total number of alters who use illicit substances with whom the respondent reported interacting during that prompt (range: 0-5), and day-level data may contain the overall sum of illicit substance–using alters across all prompts that day or the proportion of prompts to which the respondent reported interacting with any substance-using alter.

##### Location and Geographic Information Systems Data

Location data are collected once every minute using a background system process on the participant’s device. Android’s location system uses a multiple-mode sensing method to estimate location relying on a combination of WiFi, cellular triangulation, and global positioning system (GPS) satellites. The software reports accuracy as a 68% CI (1 SD) in meters; epochs with a CI greater than 100 meters were excluded. Activity spaces for participants are defined at the day level using minimum convex hulls and standard deviational ellipses (at 1, 2, and 3 SD). A minimum convex hull is a rudimentary algorithm that creates the smallest possible simple convex polygon encompassing all the points in a dataset, whereas standard deviational ellipses are mean-centered ellipses that cover 68%, 95%, and 99% of GPS data, depending on the specified SD [79]. Location activity spaces are calculated using Zone 5 of the California State Plane Coordinate System (Datum: WSG84) based on the expected geospatial distribution of data. Coordinates for activity spaces can be used to map additional data layers using geographic information systems, such as crime rate and proximity to services.

#### In-Depth Follow-Up Qualitative Interviews

**Figure 2:**
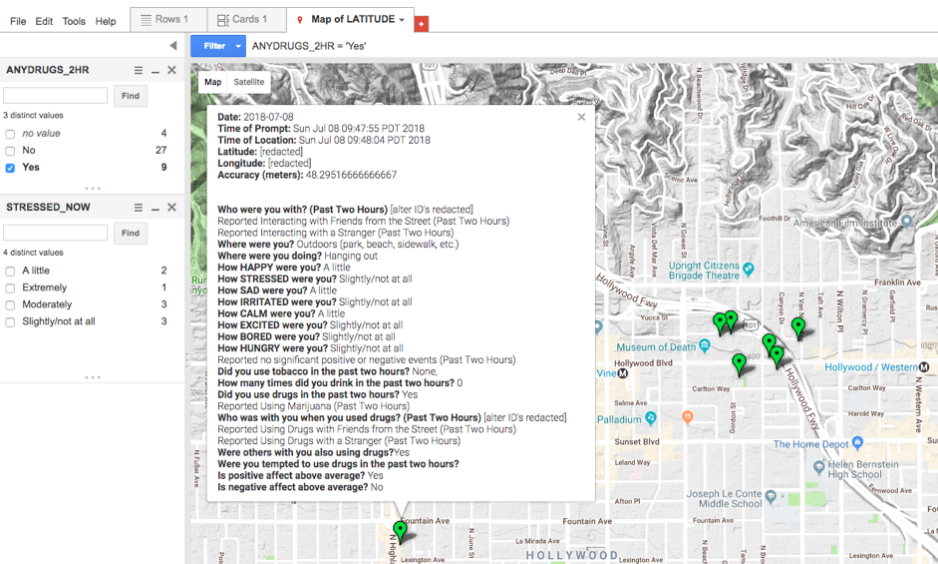
Example Google map generated from geographically explicit ecological momentary assessments responses of a participant. The exact geospatial coordinates and alter identifiers have been redacted to maintain confidentiality.

As part of the in-depth qualitative follow-up interviews, interactive geospatial maps personalized with GEMA data are shown to participants as a visual elicitation tool to explore participant risk behavior and living environment. Maps are generated in Google Maps (see example in Figure 2), and responses can be displayed on satellite imagery, a road map, or Google Street View. The maps are generated using location sensor data and momentary self-report survey data. They display where the participant traveled during the monitoring week and the locations of any drug use, stressful events, and particular areas participants may have felt positive or negative emotions (ie, happier or sadder than usual).

Interviewers first highlight responses associated with higher levels of risk behaviors (ie, drug use or risky sexual behavior) and ask participants to discuss these instances and any patterns they perceive. For example, interview questions that aim to solicit a conversation on substance use and the social contexts that affect use include: “What are your thoughts as to whether these locations influenced your using?” and “You also indicated you were/weren’t with [list any alter identified]? What role do you think this person(s) played in your using?” During the interview, participants can interact with the map, and different responses can be displayed based on any set of EMA items. Interviewers are also trained to request geospatial identifiers for daily activities, interaction with network members, and HIV risk and prevention behaviors that are not already part of the EMA response.

### Data Analysis

#### Quantitative Data Integration

EMA and daily diary data from smartphones are encrypted and then subsequently wirelessly uploaded to a secure server for further data processing. The differences in temporality between location (minute level), EMA (multiple times per day), daily diary (day level), and questionnaire (week level) data are reconciled after questionnaire data are deidentified and location and EMA data are unencrypted. Days are offset by 3 hours, ending at 2:59 am and beginning at 3:00 am to account for delayed sleep schedules in the study population. Coordinates for mean latitude and longitude during the 30-min period surrounding an EMA prompt are generated based on minute-level location data. An aggregate measure is used to limit missing data in the event that a location estimate is unavailable at the exact moment a participant answered a survey. Similarly, daily diary location data is generated using the previous day’s coordinates for mean latitude and longitude, in addition to the area of the minimum convex hull and standard deviational ellipses for that day. Daily diary data are then merged with EMA data, repeating daily diary entries across all prompts for each person-day, and questionnaire data are merged, repeating questionnaire responses across all prompts for each participant.

#### Statistical Models

All data are screened for violations of statistical assumptions, such as non-normality or outliers, and transformed to satisfy assumptions for subsequent data analyses. Pairwise correlations are used to screen for multicollinearity and exclude variables that represent similar constructs. Generalized linear models are used to examine relationships between baseline items (ie, demographics and history) with exit questionnaire outcomes. Furthermore, these models are used to predict the likelihood that an individual is assigned to supportive housing for each individual factor, as depicted in Figure 1.

Generalized linear mixed models (GLMMs) are used to address the primary aims of the study for day-level and intraday analyses and missing data analyses. Given the expected differences in contextual and psychosocial factors contingent on housing status (see Figure 1), GLMMs are conducted separately for participants in supportive housing and participants who are not housed. Model fit parameters, such as random effects and specification of variance-covariance matrices, are specified for each GLMM. All level 1 (ie, within-subject) predictors (eg, affect and hunger) are disaggregated into 2 variables through grand-mean centering and person-mean centering to examine both interindividual and intraindividual effects on the outcomes, respectively. Level 2 (ie, between-subject) factors act as covariates in all GLMMs. Depending on the outcome, contextual or psychosocial factors may act as intraday predictors, mediators, or moderators.

#### Qualitative Data Analysis and Integration

In-depth qualitative interviews are analyzed using a comparative case study analysis [80]. Case studies emphasize uniqueness in context and are used to consider complex phenomenon with interrelated influences that can exist on multiple levels (eg, individual, interpersonal, and structural). Following standard procedures for case study analysis [80,81], a case record or summary is developed for each participant using information extracted from transcripts. Transcripts are thematically analyzed using codes that help identify whether risk or protective factors are influenced by participants’ social networks, housing environment, activities participants tend to engage in, or some combination. The use of a case summary matrix that displays salient information from individual case summaries in a table format facilitates cross-case comparisons that can be used to identify broader themes [82].

Integrating qualitative and quantitative findings is done by adding significant quantitative findings to the case summary matrix to facilitate discussion of comparisons between the results. This triangulating process is used to determine the extent to which qualitative findings converge with, are complementary to, or expand upon the quantitative findings [83,84]. Discrepant findings are also noted and further considered. This information is then added to the mixed-methods matrix [85].

## Results

**Figure 3:**
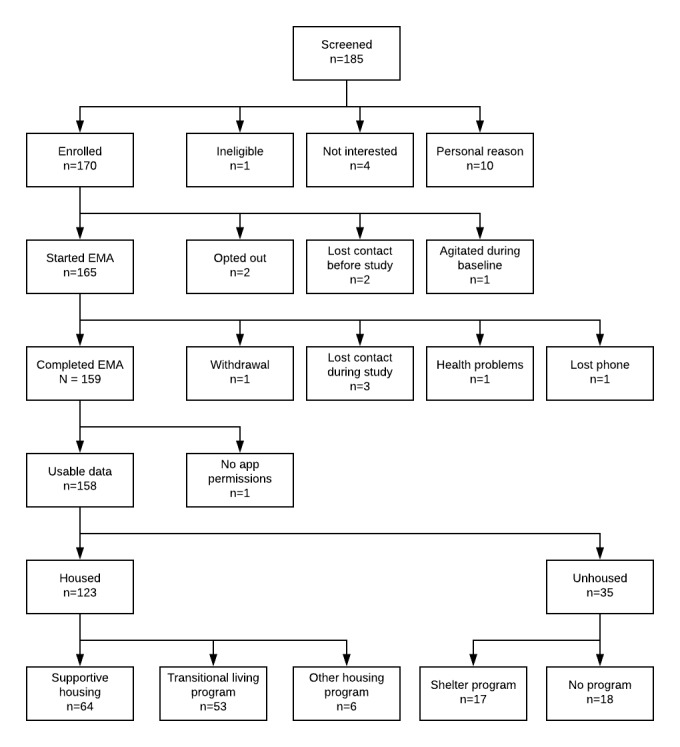
Consolidated Standards of Reporting Trials diagram for the Log My Life study. EMA: ecological momentary assessment.

Recruitment began in June of 2017. To date, 185 people have attended information sessions and were screened to participate in the study. Furthermore, 170 individuals enrolled in the study and 165 started EMA (Figure 3). Out of the 159 participants with usable data, 6 did not initially complete the protocol but then restarted the EMA and daily log component to satisfy study parameters, either by borrowing a new study phone (n=4), restarting on their personal phone after losing a study phone (n=1), or restarting on a new study phone after attempting to finish on their personal phone (n=1). Overall, 6 study phones were not returned at the end of the study, although 1 participant restarted the EMA protocol on a personal phone and the other participants opted to complete the exit questionnaire. In-depth qualitative follow-ups have been conducted with 9.5% (15/158) participants with usable data. Initial analyses of quantitative and qualitative data have begun with mixed-methods analysis planned for the near future. We expect to recruit 50 additional participants and complete analyses by September of 2019.

## Discussion

### Overview

This paper presents the protocols of a mixed-methods prospective longitudinal study designed to explore risk behavior of recently or currently homeless youth. This study is one of the few studies that have used EMA with homeless populations [20,38] and is the first, to our knowledge, that incorporates social networks with specific, identified alters in EMA, which can help improve our understanding of the role of social context. This study is also one of the first to leverage smartphone location-sensing capabilities and phone-based EMA to permit participants to report on a variety of contextual (location and physical or social surroundings) and psychosocial factors that vary throughout the day (current affect, substance use cravings, and hunger) [15]. The use of GEMA has clear methodological advantages over other approaches used in studies on homeless youth behavior, in that it can improve recall bias and ecological validity and is particularly well suited to studying substance use given its episodic nature and relation to context and current affect [15]. The use of geographic information systems also provides the ability to overlay neighborhood- or community-level data such as crime rates or density of alcohol outlets or marijuana dispensaries.

### Strengths and Limitations

We note the importance of using a mixed-methods GEMA that can provide insights into the strengths and weaknesses of traditional EMA studies [15]. EMA surveys are generally short and do not necessarily capture the richness of experiences, which is something that this study addresses through in-depth qualitative interviews that incorporate geographical considerations. Further consideration will be needed when integrating quantitative and qualitative data, especially if those data sources appear to be in conflict. Although this is the first study to use this type of mixed-methods approach to examine substance use and sexual risk behavior among homeless youth, mixed-methods studies that have included geographical data have focused on how environment affects access to public transit [86], arrhythmia in old age [87], or tobacco use [15].

Although the goal of this paper is to describe study protocols and highlight innovative aspects of the study design, we also note that the study has been successful in recruiting homeless youth who have been considered hard to engage and formerly homeless youth living in housing programs who have been understudied [88]. To date, we have had high EMA compliance and few lost phones. Further efforts are needed to understand if an incentive structure based on compliance, which has resulted in high compliance, may adversely affect data quality. Other potential challenges may include discrepancies between qualitative and quantitative reporting, missing or inaccurate geospatial data, and youth failing to admit to drug use or other risky behavior due to stigma.

### Conclusions

Despite limitations, this mixed-methods design provides rich data difficult to collect with traditional survey methodology. We know context influences health behavior [20], but we know little about the daily environments of recently homeless young adults, particularly how their movements in space and time can result in dangerous substance use or sexual activity. At a minimum, information gained from this project can inform providers of the typical risks experienced by their clients and potentially inform structural interventions in housing programs. Geospatial data could point to *hotspots* (see Veldhuizen et al [89]) or specific risky environments in which young adults often interact. Furthermore, ecologically valid methods allow researchers to approximate what might be occurring in real time and bring us one step closer to identifying leverage points where intervention might be particularly fruitful. The combination of EMA and geospatial data can greatly enhance the development of ecological momentary interventions or just-in-time adaptive interventions [90,91]. Theoretically, these interventions harness the power of mobile phone technology, particularly geospatial sensors and app-based momentary prompts, to intervene at just the right time to shift an individual’s behavior. Mobile interventions are potentially affordable solutions to intervening among populations that are typically hard to reach. We now have the technology to deliver preventive interventions in situ, but we do not yet fully understand the dynamic nature of health risk behaviors [92]. We hope that studies such as this can begin to untangle this complexity.

## Appendix

Multimedia Appendix 1 Ecological momentary assessment (EMA) items.

Multimedia Appendix 2 Sample smartphone app screen images.

Multimedia Appendix 3 Daily log items.

## Abbreviations

EMA: ecological momentary assessment
GEMA: geographically explicit ecological momentary assessments
GLMM: generalized linear mixed model
GPS: global positioning system
LML: Log My Life
