# Supplementary material for: Investigating Health Risk Environments in Housing Programs for Young Adults: Protocol for a Geographically Explicit Ecological Momentary Assessment Study
Source: JMIR Res Protoc. 2019 Jan 10;8(1):e12112. doi: 10.2196/12112 (PMC6329898; doi:10.2196/12112)
Supplement: Multimedia Appendix 1 [file resprot_v8i1e12112_app1.pdf]

Multimedia Appendix 1: Table of Ecological Momentary Assessment (EMA) items.[a]

Variable	Item	Response options
**(A) Social context**	(1) Over the past 2 hours, who have you interacted with in any way?<br>(Check all)	• [Alter 1]<br>• [Alter 2]<br>• [Alter 3]<br>• [Alter 4]<br>• [Alter 5]<br>• Someone else not listed here<br>• I have not interacted with anyone
**(A) Social context**	[Display if “someone else not listed here is selected for A-1]<br>(1.1.) Over the past 2 hours, who else have you interacted with?<br>(Check all)	• Friends from home/before you were homeless<br>• Friends or peers from the street or an agency<br>• Family (biological or foster)<br>• Romantic/sexual partner<br>• Case worker or agency staff/volunteer<br>• People from work or school<br>• Law enforcement (police, security, etc.)<br>• Someone I don’t know well/random person
**(B) Location**	(1) Where are you currently?	• My apartment/residence<br>• Someone else’s residence<br>• In transit (bus, car, etc.)<br>• Outdoors (park, beach, sidewalks, etc.)<br>• School or work<br>• Social service agency (drop-in, shelter, DPSS, etc.)<br>• Place of business (restaurant, bar, mall, etc.)<br>• Other
**(C) Activity**	(1) Just before the phone went off, what was the main thing you were doing?	• Going somewhere<br>• Hanging out<br>• Working/job/school<br>• Meal/eating food<br>• Sleeping/resting<br>• Appointment/meeting<br>• Other
**(D) Affect**	(1) Just before the phone went off, how HAPPY were you feeling?<br>(2) Just before the phone went off, how STRESSED were you feeling?<br>(3) Just before the phone went off, how SAD/DEPRESSED were you feeling?<br>(4) Just before the phone went off, how IRRITATED were you feeling?<br>(5) Just before the phone went off, how CALM/RELAXED were you feeling?	• Slightly/not at all<br>• A little<br>• Moderately<br>• Quite a bit<br>• Extremely

Variable	Item	Response options
	(6) Just before the phone went off, how EXCITED were you feeling?<br>(7) Just before the phone went off, how BORED were you feeling?	
**(E) Hunger**	(1) Just before the phone went off, how HUNGRY were you feeling?	• Slightly/not at all<br>• A little<br>• Moderately<br>• Quite a bit<br>• Extremely
**(F) Important event**	(1) Over the past 2 hours, did any of these things happen to you?<br>(Check all)	• I felt threatened or harassed<br>• Verbal fight or argument<br>• Physical fight<br>• I got injured or became ill<br>• Received bad news about something important<br>• Received good news about something important<br>• Interaction with security/law enforcement<br>• None of the above
**(G) Tobacco**	(1) Over the past 2 hours, have you used…<br>(Check all)	• I have not used tobacco<br>• Smoked tobacco (cigarettes, cigars/cigarillos, pipes, hookah, etc.)<br>• E-cigarettes/vaped tobacco<br>• Chewing tobacco/dip<br>• A tobacco product not listed here
**(H) Alcohol**	(1) Over the past 2 hours, how many alcoholic drinks did you have?	• 0<br>• 1<br>• 2<br>• 3<br>• 4<br>• 5 or more
**(H) Alcohol**	[Display if “0” is NOT selected for H-1]<br>(1.1.) Where were you when you used alcohol?<br>(Check all)	• My apartment/residence<br>• Someone else’s residence<br>• In transit (bus, car, etc.)<br>• Outdoors (park, beach, sidewalks, etc.)<br>• School or work<br>• Social service agency (drop-in, shelter, DPSS, etc.)<br>• Place of business (restaurant, bar, mall, etc.)<br>• Other
**(H) Alcohol**	[Display if “0” is NOT selected for H-1]<br>(1.2.) Who were you with when you used alcohol?<br>(Check all)	• [Alter 1]<br>• [Alter 2]<br>• [Alter 3]<br>• [Alter 4]<br>• [Alter 5]<br>• Someone else not listed here

Variable	Item	Response options
		• Nobody
**(H) Alcohol**	[Display if “Someone else not listed here” is selected for H-1.2.]<br>(1.2.1.) Who else were you with when you used alcohol?<br>(Check all)	• Friends from home/before you were homeless<br>• Friends or peers from the street or an agency<br>• Family (biological or foster)<br>• Romantic/sexual partner<br>• People from work or school<br>• Someone I don’t know well/random person
**(H) Alcohol**	[Display if “Nobody” is NOT selected for H-1.2.]<br>(1.2.2.) Were any of the people with you using alcohol?	• Yes<br>• No<br>• Not sure
**(I) Drugs**	(1) Over the past 2 hours, have you used any drugs?	• Yes<br>• No
**(I) Drugs**	[Display if “Yes” is selected for I-1]<br>(1.1.) Which drugs did you use over the past 2 hours?<br>(Check all)	• Marijuana<br>• Meth<br>• Ecstasy/ MDMA/ “Molly”<br>• Synthetic marijuana (K2, Spice, etc.)<br>• Hallucinogens/ psychedelics<br>• Prescription drugs, not as prescribed (Rx cough syrup, Oxycontin, Xanax, etc.)<br>• Heroin<br>• Other
**(I) Drugs**	[Display if “Other” is selected for I-1.1.]<br>(1.1.1.) What other drug did you use? (Please specify)	[Text Entry]
**(I) Drugs**	[Display if “Yes” is selected for I-1]<br>(1.2.) Where were you when you used drugs?<br>(Check all)	• My apartment/residence<br>• Someone else’s residence<br>• In transit (bus, car, etc.)<br>• Outdoors (park, beach, sidewalks, etc.)<br>• School or work<br>• Social service agency (drop-in, shelter, DPSS, etc.)<br>• Place of business (restaurant, bar, mall, etc.)<br>• Other
**(I) Drugs**	[Display if “Yes” is selected for I-1]<br>(1.3.) Who were you with when you used drugs?<br>(Check all)	• [Alter 1]<br>• [Alter 2]<br>• [Alter 3]<br>• [Alter 4]<br>• [Alter 5]<br>• Someone else not listed here<br>• Nobody
**(I) Drugs**	[Display if “Someone else not listed here” is selected for I-1.3.]<br>(1.3.1.) Who else were you with when you used	• Friends from home/before you were homeless<br>• Friends or peers from the street or an

Variable	Item	Response options
	drugs?<br>(Check all)	agency<br>• Family (biological or foster)<br>• Romantic/sexual partner<br>• People from work or school<br>• Someone I don't know well/random person
**(I) Drugs**	[Display if "Nobody" is NOT selected for I-1.3.]<br>(1.3.2.) Were any of the people you were with using drugs?	• Yes<br>• No<br>• Not sure
**(J) Craving**	[Display variable J only if "0" is selected for H-1 AND "No" is selected for I-1]<br>(1) Over the past 2 hours, did you think about using drugs or alcohol?	• Yes<br>• No
**(J) Craving**	[Display if "Yes" is selected for J-1]<br>(1.1.) Where were you when you thought about using drugs or alcohol?<br>(Check all)	• My apartment/residence<br>• Someone else's residence<br>• In transit (bus, car, etc.)<br>• Outdoors (park, beach, sidewalks, etc.)<br>• School or work<br>• Social service agency (drop-in, shelter, DPSS, etc.)<br>• Place of business (restaurant, bar, mall, etc.)<br>• Other
**(J) Craving**	[Display if "Yes" is selected for J-1]<br>(1.2.) Who were you with when you thought about using drugs or alcohol?<br>(Check all)	• [Alter 1]<br>• [Alter 2]<br>• [Alter 3]<br>• [Alter 4]<br>• [Alter 5]<br>• Someone else not listed here<br>• Nobody
**(J) Craving**	[Display if "Someone else not listed here" is selected for J-1.2.]<br>(1.2.1.) Who else were you with when you thought about using drugs or alcohol?	• Friends from home/before you were homeless<br>• Friends or peers from the street or an agency<br>• Family (biological or foster)<br>• Romantic/sexual partner<br>• People from work or school<br>• Someone I don't know well/random person
**(J) Craving**	[Display if "Nobody" is NOT selected for J-1.2.]<br>(1.2.2.) When you thought about using drugs or alcohol, were any of the people you were with using drugs or alcohol?	• Yes<br>• No<br>• Not sure

[a]Names entered during the baseline survey for alters 1-5 are piped in as response options for each participant.
