# Supplementary material for: Investigating Health Risk Environments in Housing Programs for Young Adults: Protocol for a Geographically Explicit Ecological Momentary Assessment Study
Source: JMIR Res Protoc. 2019 Jan 10;8(1):e12112. doi: 10.2196/12112 (PMC6329898; doi:10.2196/12112)
Supplement: Multimedia Appendix 2 [file resprot_v8i1e12112_app2.pdf]

Multimedia Appendix 2: Sample smartphone application screen images.

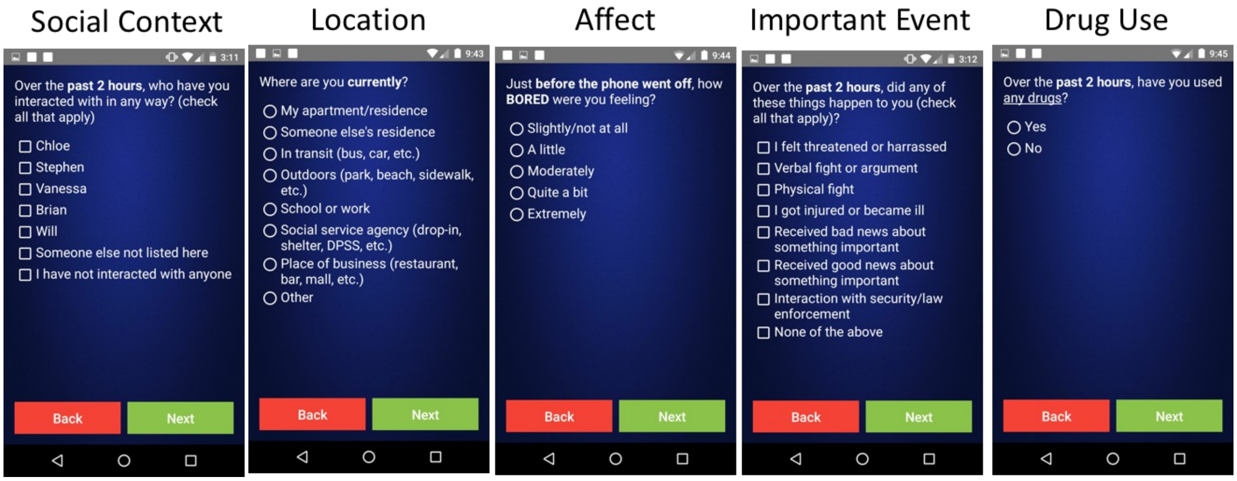

Note. “Chole” or “Will” are examples of participant-specific alter names automatically added into question text.
