# Supplementary material for: Investigating Health Risk Environments in Housing Programs for Young Adults: Protocol for a Geographically Explicit Ecological Momentary Assessment Study
Source: JMIR Res Protoc. 2019 Jan 10;8(1):e12112. doi: 10.2196/12112 (PMC6329898; doi:10.2196/12112)
Supplement: Multimedia Appendix 3 [file resprot_v8i1e12112_app3.pdf]

Multimedia Appendix 3: Table of Daily log items.

Variable	Item	Response options
**(A) Sleep**	(1) What time did you wake-up today?	[Possible times in 15 min intervals on scroll wheel]<br>• I did not sleep last night
**(A) Sleep**	[Display if "I did not sleep last night" is NOT selected for A-1.]<br>(1.1.) What time did you go to sleep yesterday?	[Possible times in 15 min intervals on scroll wheel]
**(A) Sleep**	[Display if "I did not sleep last night" is NOT selected for A-1.]<br>(1.2.) Where did you sleep last night?	• My own apartment/residence<br>• Home of someone I know (family, friend, partner, etc.)<br>• Stranger's home/residence<br>• Shelter/transitional living program<br>• Hotel/motel<br>• Abandoned building, squat, public transit, or other place inside<br>• Street, park, bench, roof, or other place outside<br>• Car, RV, van, or other vehicle<br>• Other, please specify
**(A) Sleep**	[Display if "Other" is selected for A-1.2.]<br>(1.2.1.) What other place did you sleep last night?	[Text entry]
**(A) Sleep**	[Display if "I did not sleep last night" is NOT selected for A-1.]<br>(1.3.) Last night, my sleep quality was:	• Very poor<br>• Poor<br>• Fair<br>• Good<br>• Very good
**(B) Social Context**	(1) At any point yesterday, did you interact with any of these people in any way? (Check all)	• [Alter 1]<br>• [Alter 2]<br>• [Alter 3]<br>• [Alter 4]<br>• [Alter 5]<br>• I did not interact with any of these people yesterday
**(C) Substances**	(1) At any point yesterday, did you use any of these substances? (Check all)	• Alcohol<br>• Marijuana<br>• Synthetic marijuana (K2, spice, etc.)<br>• Meth<br>• Prescription drugs, not as prescribed (Rx cough syrup, Oxycontin, Xanax, etc.)<br>• Other illicit drugs<br>• I did not use any drugs yesterday
**(C) Substances**	[Display if "Other illicit drug" is selected for C-1.]<br>(1.1.) What other illicit drug(s) did you use yesterday? (Check all)	• Ecstasy/ MDMA/ "Molly"<br>• Hallucinogens/ psychedelics<br>• Heroin<br>• Cocaine or crack<br>• Something else not listed here

Variable	Item	Response options
**(C) Substances**	[Display if “Something else not listed here” is selected for C-1.1.]<br>(1.1.1.) You indicated that you used something else that wasn’t listed. How did you use that/those other drug(s) yesterday? (Check all)	• Swallowed<br>• Smoked<br>• Snorted<br>• Injected<br>• Other
**(C) Substances - Alcohol**	[Display if “Alcohol” is selected for C-1.]<br>(1.2.) How many alcoholic drinks did you have yesterday?<br>Note: “One drink” is:<br>-a 12-oz beer<br>-1/5 of a 40-oz bottle of malt liquor<br>-a 5-oz glass of wine<br>-a 1.5 oz “shot” of 80-proof liquor	[Number picker; range = 0-99]
**(C) Substances - Alcohol**	[Display if response is ≥ 4/5 (4 for women/5 for men) for C-1.2.]<br>(1.2.1.) At any point yesterday, did you drink 4/5 [4 for women/5 for men] or more drinks in less than a 2-hour period?	• Yes<br>• No<br>• Not sure
**(C) Substances - Alcohol**	[Display if “Alcohol” is selected for C-1.]<br>(1.3.) At any point yesterday, did you drink alcohol with any of these people?<br>(Check all)	• [Alter 1]<br>• [Alter 2]<br>• [Alter 3]<br>• [Alter 4]<br>• [Alter 5]<br>• I did not drink alcohol with any of these people yesterday
**(C) Substances - Marijuana**	[Display if “Marijuana” is selected for C-1.]<br>(1.4.) How many times did you use marijuana or marijuana products yesterday?	[Number picker; range = 0-99]
**(C) Substances - Marijuana**	[Display if “Marijuana” is selected for C-1.]<br>(1.5.) At any point yesterday, did you use marijuana concentrates? (hashish, hash oil, “dabs,” etc.)	• Yes<br>• No<br>• Not sure
**(C) Substances - Marijuana**	[Display if “Marijuana” is selected for C-1.]<br>(1.6.) At any point yesterday, did you use marijuana or marijuana products with any of these people? (Check all)	• [Alter 1]<br>• [Alter 2]<br>• [Alter 3]<br>• [Alter 4]<br>• [Alter 5]<br>• I did not use marijuana or marijuana products with any of these people yesterday
**(C) Substances – Synthetic Marijuana**	[Display if “Synthetic marijuana” is selected for C-1.]<br>(1.7.) How many times did you use synthetic marijuana yesterday? (K2, spice, fake weed, etc.)	[Number picker; range = 0-99]
**(C) Substances – Synthetic Marijuana**	[Display if “Synthetic marijuana” is selected for C-1.]<br>(1.8.) At any point yesterday, did you use synthetic marijuana with any of these people? (Check all)	• [Alter 1]<br>• [Alter 2]<br>• [Alter 3]<br>• [Alter 4]<br>• [Alter 5]

Variable	Item	Response options
		• I did not use synthetic marijuana with any of these people yesterday
**(C) Substances – Meth**	[Display if “Meth” is selected for C-1.]<br>(1.9.) How many times did you use meth yesterday?	[Number picker; range = 0-99]
**(C) Substances – Meth**	[Display if “Meth” is selected for C-1.]<br>(1.10.) How did you use meth yesterday? (Check all)	• Smoked<br>• Snorted<br>• Injected<br>• Swallowed
**(C) Substances – Meth**	[Display if “Meth” is selected for C-1.]<br>(1.11.) At any point yesterday, did you use meth with any of these people?<br>(Check all)	• [Alter 1]<br>• [Alter 2]<br>• [Alter 3]<br>• [Alter 4]<br>• [Alter 5]<br>• I did not use meth with any of these people yesterday
**(C) Substances – Prescription Drugs**	[Display if “Prescription drugs…not prescribed…” is selected for C-1.]<br>(1.12.) How many times did you use prescription drugs that were not prescribed, or in higher doses than prescribed, yesterday?	[Number picker; range = 0-99]
**(C) Substances – Prescription Drugs**	[Display if “Prescription drugs…not prescribed…” is selected for C-1.]<br>(1.13.) How did you use prescription drugs, not as prescribed yesterday? (Check all)	• Swallowed<br>• Snorted<br>• Injected<br>• Smoked
**(C) Substances – Prescription Drugs**	[Display if “Prescription drugs…not prescribed…” is selected for C-1.]<br>(1.14.) At any point yesterday, did you use prescription drugs, not as prescribed with any of these people? (Check all)	• [Alter 1]<br>• [Alter 2]<br>• [Alter 3]<br>• [Alter 4]<br>• [Alter 5]<br>• I did not use prescription drugs, not as prescribed with any of these people yesterday
**(C) Substances – Ecstasy**	[Display if “Ecstasy/ MDMA/ Molly” is selected for C-1.]<br>(1.15.) How many times did you use Ecstasy/ MDMA/ “Molly” yesterday?	[Number picker; range = 0-99]
**(C) Substances – Ecstasy**	[Display if “Ecstasy/ MDMA/ Molly” is selected for C-1.]<br>(1.16.) How did you use Ecstasy/ MDMA/ “Molly” yesterday? (Check all)	• Swallowed<br>• Snorted<br>• Injected
**(C) Substances – Ecstasy**	[Display if “Ecstasy/ MDMA/ Molly” is selected for C-1.]<br>(1.17.) At any point yesterday, did you use Ecstasy/ MDMA/ “Molly” with any of these people? (Check all)	• [Alter 1]<br>• [Alter 2]<br>• [Alter 3]<br>• [Alter 4]<br>• [Alter 5]<br>• I did not use Ecstasy/ MDMA/ “Molly” with any of these people yesterday
**(C) Substances –**	[Display if “Hallucinogens/ psychedelics” is	[Number picker; range = 0-99]

Variable	Item	Response options
**Hallucinogens**	selected for C-1.]<br>(1.18.) How many times did you use hallucinogens/ psychedelics (LSD/acid, "shrooms", etc.) yesterday?	
**(C) Substances – Hallucinogens**	[Display if "Hallucinogens/ psychedelics" is selected for C-1.]<br>(1.19.) At any point yesterday, did you use hallucinogens/ psychedelics (LSD/acid, "shrooms", etc.) with any of these people?<br>(Check all)	• [Alter 1]<br>• [Alter 2]<br>• [Alter 3]<br>• [Alter 4]<br>• [Alter 5]<br>• I did not use hallucinogens/ psychedelics with any of these people yesterday
**(C) Substances – Heroin**	[Display if "Heroin" is selected for C-1.]<br>(1.20.) How many times did you use heroin yesterday?	[Number picker; range = 0-99]
**(C) Substances – Heroin**	[Display if "Heroin" is selected for C-1.]<br>(1.21.) How did you use heroin yesterday?	• Snorted<br>• Injected<br>• Smoked
**(C) Substances – Heroin**	[Display if "Heroin" is selected for C-1.]<br>(1.22.) At any point yesterday, did you use heroin with any of these people?<br>(Check all)	• [Alter 1]<br>• [Alter 2]<br>• [Alter 3]<br>• [Alter 4]<br>• [Alter 5]<br>• I did not use heroin with any of these people yesterday
**(C) Substances – Cocaine**	[Display if "Cocaine or crack" is selected for C-1.]<br>(1.23.) You indicated that you used cocaine or crack yesterday. Which type(s) did you use?	• Powdered cocaine<br>• Crack or freebase cocaine<br>• Both
**(C) Substances – Cocaine**	[Display if "Cocaine or crack" is selected for C-1.]<br>(1.24.) How many times did you use cocaine or crack yesterday? (Check all)	[Number picker; range = 0-99]
**(C) Substances – Cocaine**	[Display if "Cocaine or crack" is selected for C-1.]<br>(1.25.) How did you use cocaine or crack yesterday? (Check all)	• Orally (swallowed, chewed, rubbed on gums, etc.)<br>• Smoked<br>• Snorted<br>• Injected
**(C) Substances – Cocaine**	[Display if "Cocaine or crack" is selected for C-1.]<br>(1.26.) At any point yesterday, did you use cocaine or crack with any of these people?<br>(Check all)	• [Alter 1]<br>• [Alter 2]<br>• [Alter 3]<br>• [Alter 4]<br>• [Alter 5]<br>• I did not use cocaine or crack with any of these people yesterday
**(D) Sexual Activity**	(1) How many people did you have vaginal or anal sex with yesterday?	• 0<br>• 1<br>• 2<br>• 3<br>• 4 or more

Variable	Item	Response options
**(D) Sexual Activity**	[D-1.1. – D-1. are displayed if response is > 0 for D-1 and looped based on # of partners.]<br>(1.1.) Please provide initials or a nickname for the first person you had vaginal or anal sex with yesterday:	[Text entry]
**(D) Sexual Activity**	(1.2.) How would you describe [Partner 1]?	• Serious partner (husband, wife, life partner, girlfriend, boyfriend, etc.)<br>• Casual partner (hookup, friends with benefits, one-night stand, etc.)
**(D) Sexual Activity**	(1.3.) How long have you been engaged in a sexual relationship with [Partner 1]?<br>(in total)	• 1 day or less<br>• 1-30 days<br>• 1-6 months<br>• 6-12 months<br>• 1-3 years<br>• More than 3 years
**(D) Sexual Activity**	(1.4.) What is [Partner 1]'s gender identity?	• Male<br>• Female<br>• Trans male/ Trans man<br>• Trans female/ Trans woman<br>• Genderqueer/ Gender non-conforming<br>• Different identity (please specify_____)
**(D) Sexual Activity**	[Do not display if both respondent and partner are women]<br>(1.4.1.) At any point while having sex with [Partner 1], did you have vaginal or anal sex without a condom?	• No<br>• Yes, both anal and vaginal sex without a condom<br>• Yes, vaginal without a condom only<br>• Yes, anal without a condom only
**(D) Sexual Activity**	[Display if "No" is NOT selected for D-1.4.1.]<br>(1.4.1.1.) Did you know [Partner 1]'s HIV status before having vaginal or anal sex without a condom with them?	• Yes<br>• No
**(D) Sexual Activity**	[Display if "No" is NOT selected for D-1.4.1.]<br>(1.4.1.2.) Did you drink alcohol or use drugs before you had sex (vaginal or anal sex) with [Partner 1]?	• Yes<br>• No
**(D) Sexual Activity**	(1.5.) Where were you when you engaged in sex with [Partner 1}? (Check all)	• My home/ residence<br>• The home of someone I know<br>• The home of someone I don't know or barely know<br>• Hotel/ motel<br>• Outside (park, bench)<br>• Tent or improvised shelter<br>• Car, RV, or other vehicle<br>• Abandoned building or squat<br>• Shelter or drop-in<br>• Other (please specify_____)
**(D) Sexual Activity**	(2) At any point yesterday, did you trade any type of sex (oral, vaginal, or anal) for: (Check all)	• I did not trade sex<br>• Money<br>• Drugs

Variable	Item	Response options
		• A place to stay<br>• Food or meals<br>• Something else
**(D) Sexual Activity**	[Display if "I did not trade sex" is NOT selected for D-2.]<br>(2.1.) Where were you when you traded sex for money, drugs, a place to stay, food or meals, or something else? (Check all)	• My home/ residence<br>• The home of someone I know<br>• The home of someone I don't know or barely know<br>• Hotel/ motel<br>• Outside (park, beach)<br>• Tent or improvised shelter<br>• Car, RV, or other vehicle<br>• Abandoned building or squat<br>• Shelter or drop-in<br>• Other (please specify______)

[a]Names entered during the baseline survey for alters 1-5 are piped in as response options for each participant.
